# Letter response: Intra-familial phenotype variability in Late-Onset Tay-Sachs disease

**DOI:** 10.5334/tohm.756

**Published:** 2023-02-22

**Authors:** Giulietta Maria Riboldi, Heather Lau

**Affiliations:** 1 The Marlene and Paolo Fresco Institute for Parkinson’s Disease and Movement Disorders, New York University Langone Health, New York, NY, United States of America; 2 Department of Neurology, New York University Langone Health, New York, NY, United States of America; 3 Yale University, Department of Internal Medicine, United States of America

**Keywords:** Late-Onset Tay-Sachs Disease, phenotype, family history, intra-familial, siblings

We thank Lefter and Ryan for the interesting comment to our manuscript. We agree with their observation in regards of the interfamilial phenotypic variability within subjects with Late Onset Tay-Sachs (LOTS) disease.

In order to strength this observation, we further reviewed the cases reported in the literature and that were included in our publication. As we reported in our manuscript, a positive family history of LOTS was present in 58.8% of subjects presenting with a mainly neuromuscular phenotype, 75% of subjects with a predominant cerebellar phenotype, 37.5% of subjects with prevalent psychiatric manifestation, and 83.3% of subjects with a predominant stuttering phenotype [1].

As noted in Navon et al., 1986, one of the initial works reporting familial cases of LOTS stated that “affected relatives of the same family had a rather uniform clinical picture” [2]. However, as the same authors comment later on in the manuscript and as suggested by Lefter and Ryan (2022), this has not been necessarily confirmed in the following literature [2].

Consistently with the above observations, we reviewed the manuscripts where the clinical history of probands and their relatives was detailed and we found a spectrum of clinical presentations within families. In particular, some of the works reported very similar phenotypic presentation (i.e. predominant neurological and psychiatric manifestations) as well as a similar progression and severity of symptoms over time within affected members of the same family [2,3,4,5,6,7]. In some families, the phenotype was similar (i.e. predominant cerebellar or stuttering phenotype at presentation) but the age of onset within different individuals in the same family and the progression of the symptoms was different [3,8]. Other authors described, instead, a significant intra-familial variability in the reported cases, both in terms of clinical presentation and timeline of symptom onset [2,3,9,10,11,12,13].

The genotype was not consistently reported for all these cases, so it is not possible to speculate about a role of different genetic variants in the context of intra-familial variability. Interestingly, Willner et al., 1981 reported one family with two affected identical twins presenting an overlapping phenotype and disease progression, while the two other affected siblings in the same family presented a similar clinical phenotype but a different rate of progression of their symptoms [3].

We thank Lefter and Ryan for their clarification regarding this point raised by our manuscript and we believe that these additional observations will further enrich the content of our paper and our understanding of LOTS.
